# High-Shear Wet Granulation of SMEDDS Based on Mesoporous Carriers for Improved Carvedilol Solubility

**DOI:** 10.3390/pharmaceutics14102077

**Published:** 2022-09-29

**Authors:** Mila Kovačević, Ilija German Ilić, Katarina Bolko Seljak, Alenka Zvonar Pobirk

**Affiliations:** Department of Pharmaceutical Technology, Faculty of Pharmacy, University of Ljubljana, 1000 Ljubljana, Slovenia

**Keywords:** SMEDDS, mesoporous carriers, wet granulation, high-shear granulator

## Abstract

Mesoporous carriers are a convenient choice for the solidification of self-microemulsifying drug delivery systems (SMEDDS) designed to improve the solubility of poorly water-soluble drugs. They are known for high liquid load capacity and the ability to maintain characteristics of dry, free-flowing powders. Therefore, five different mesoporous carriers were used for the preparation of carvedilol-loaded SMEDDS granules by wet granulation methods—in paten (manually) and using a high-shear (HS) granulator. Granules with the highest SMEDDS content (63% and 66% of total granules mass, respectively) and suitable flow properties were obtained by Syloid^®^ 244FP and Neusilin^®^ US2. SMEDDS loaded granules produced by HS granulation showed superior flow characteristics compared to those obtained manually. All SMEDDS granules exhibited fast in vitro release, with 93% of carvedilol releasing from Syloid^®^ 244FP-based granules in 5 min. Upon compaction into self-microemulsifying tablets, suitable tablet hardness and very fast disintegration time were achieved, thus producing orodispersible tablets. The compaction slightly slowed down the carvedilol release rate; nevertheless, upon 1 h (at pH 1.2) or 4 h (at pH 6.8) of in vitro dissolution testing, the amount of released drug was comparable with granules, confirming the suitability of orodispersible tablets for the production of the SMEDDS loaded single unit oral dosage form.

## 1. Introduction

During the 21st century, great progress in the field of pharmaceutical science has been achieved, yet the formulation of poorly water soluble active pharmaceutical ingredients (APIs) remains a leading challenge within pharmaceutical technology. Since biopharmaceutical characteristics of pharmaceutical product strongly depend on the API aqueous solubility, strategies for its enhancement are of vital importance in formulation development. Among different approaches, self-(micro)emulsifying drug delivery systems (SMEDDS) are known for large solubilization capacity and therefore have an ability to keep the API dissolved throughout its transit via gastro-intestine, resulting in enhanced oral bioavailability [1].

SMEDDS also proved to be successful for the solubility improvement of carvedilol, a BCS class II drug [2,3,4]. By non-selectively blocking β adrenergic receptors, the drug inhibits the effect of the sympathetic nervous system, while antagonizing α_1_ adrenergic receptors causes a vasodilatation effect that overall legitimatizes its use in the treatment of certain cardiovascular diseases. Apart from SMEDDS, the solubility enhancement was also achieved by amorphous solid dispersion preparation using porous silica [5,6]. Nevertheless, as the substrate for CYP enzymes in the liver, incorporation into SMEDDS formulation gives an additional benefit due to the reduction in the first-pass metabolism effect [1]. Still, one disadvantage of SMEDDS is that they are present in liquid form, while singe-dose solid dosage forms (e.g., tablets) are known to achieve better patient compliance [7,8]. One solution is transformation of SMEDDS to solids, with the goal to improve the processability and stability of the final product. Since Sandimmune Neoral^®^ was launched in 1994, no other solid dosage forms of SMEDDS besides hard or soft capsules have entered the market, despite numerous advantages and extensive research in this field [8]. In the last decade, worldwide researchers have extensively investigated different solidification techniques, with the modest contribution of our research group as well [2,3,9,10,11]. So far, various solidification technologies have been investigated among SMEDDS, such as adsorption to solid carriers, granulation, pelletization and pellet coating, spray drying and hot-melt extrusion [3,10,11,12,13,14,15,16,17,18]. Among them, wet granulation is one of the most interesting and promising techniques, especially considering its availability in the pharmaceutical industry.

When using fluid-bed equipment, the resulting granules usually have a porous structure and irregular shape, as particles are in a fluidized state due to upward airflow. On the contrary, with the use of high-shear equipment, the resulting granules usually have higher bulk and tapped densities as well as a more round/spherical shape, due to high-shear forces during processing. Therefore, differences between final granules are also manifested in the flow properties, depending on the granules size, size distribution, shape, bulk and tapped densities. Moreover, good flowability is critical for successful further processing, such as tableting or capsule filling [2]. Research has shown that optimal SMEDDS granules produced by using fluid-bed equipment exhibited passable and poor flow properties. The authors explained that irregular shape and high SMEDDS loading (46% and 65% *m*/*m*) contributed to such a result. That is, in fluid-bed wet granulation, the dispersion droplets are in contact with carrier particles for a very short time as they dry under a warm airflow. Therefore, there is not enough time for deeper penetration into the pores, as SMEDDS are also deposited on the surface [2]. In another study [14], the authors investigated the influence of high-shear wet granulation process parameters on the final characteristics of self-emulsifying granules with simvastatin. During the process, the drug-loaded microemulsion was dripped onto a powder mixture containing microcrystalline cellulose, lactose and 3% povidone. The produced granules were further characterized with determination of the mean diameter, shape, disintegration time and drug dissolution rate. However, the product flow properties were not evaluated [14]. The remaining challenge in wet granulation with SMEDDS is also to obtain a high liquid load, as it correlates with high API content in granules. The flow properties of such granules tend to be poor, due to lipidic consistency, resulting in a sticky product. However, good flowability is vital for further product processing, as it enables efficient tableting and capsule filling on an industrial scale.

Mesoporous carriers (pore size 2–50 nm) are known for their high liquid load capacity and can adsorb a high amount of liquid, while maintaining the characteristics of dry, free-flowing powders. Therefore, having these properties, they present a huge potential as carrier excipients for SMEDDS solidification [19,20,21,22]. Neusilin^®^ US2 (magnesium aluminometasilicate), as an example of such a carrier, was used in fluid-bed SMEDDS granulation, where the product was further compressed into fast-releasing tablets. Syloid^®^ 244 FP (silica-based mesoporous carrier) was also studied, although efficient fluidization of the carrier could not be obtained, as the filter bags clogged due to the small particle size of the carrier [2]. In such cases, high-shear (HS) granulation offers a suitable alternative, as the carrier is placed inside the bowl and is mixed by blades, rather than being dispersed in the chamber of the fluid-bed granulator. Thus, in the study covering different SMEDDS solidification technologies [11], high-shear equipment was used to produce SMEDDS powders, based on Neusilin^®^ US2 as well. The results demonstrated low liquid load capacity (carrier:SMEDDS ratio of 1:1), fair-to-passable flow properties, and retained self-emulsifying properties. As it was a part of the preliminary research with actual focus on the spray-drying technique, the HS granulation technology has not yet been extensively studied for the solidification of SMEDDS with mesoporous carriers, and further systematic research is required for its improvement and final industrial application.

Therefore, the purpose of the present study was to investigate the relevant technological and biopharmaceutical characteristics of SMEDDS granules containing carvedilol, a poorly water-soluble model drug. Five different mesoporous carriers were loaded with liquid SMEDDS, with the aim of obtaining granules with high SMEDDS content and good flow properties. Such a balance is difficult to achieve, but still necessary for the further production of self-microemulsifying prototype tablets. Thus, the most promising carriers were chosen for scale-up using HS equipment for wet granulation, with the goal to use them for compaction into SMEDDS tablets with adequate mechanical characteristics and a fast in vitro drug release profile.

## 2. Materials and Methods

### 2.1. Materials

Carvedilol (CTX Life Sciences Ltd., Gujarat, India) was used as a model drug.

Liquid SMEDDS was composed of Capmul^®^ MCM EP/NF (mono-diglyceride of medium chain fatty acids, Abitec Corporation, Columbus, OH, USA), refined castor oil (Ph. Eur. Grade, Caesar & Loretz GmbH, Hilden, Germany), Kollisolv^®^ PEG E 400 (Sigma-Aldrich, St. Louis, MO, USA) and Kolliphor^®^ RH 40 (polyoxyl 40 hydrogenated castor oil, Sigma-Aldrich, USA).

Five different mesoporous carriers were used for SMEDDS granulation: Sysloid^®^ 244 FP (silica with average particle size 3.5 μm, Grace GmbH & Co. KG, Worms, Germany), Fujicalin^®^ SG (SA) (anhydrous calcium hydrogen phosphate with mean particle size 120 μm, Fuji Chemical Industries Co. Ltd., Toyama, Japan), Neusilin^®^ US2 (amorphous magnesium aluminometasilicate with mean particle size 106 μm, Fuji Chemical Industries Co. Ltd., Japan), Syloid^®^ XDP 3050 (silica with average particle size 50 μm, Grace GmbH & Co. KG, Germany), and Aeroperl^®^ 300 (fumed silica with average particle size 20–60 μm, EVONIK, Essen, Germany). Povidone K25 (Kollidon^®^ 25) was used as a binder in granulation dispersion (GD).

For SMEDDS tablet production, additional excipients were used, specifically copovidone VA64 (Kollidon^®^ VA64, BASF, Ludwigshafen, Germany) as a binder, crosscarmellose sodium (Ac-Di-Sol^®^, FMC BioPolymer, Philadelphia, PA, USA) as a disintegrant, microcrystalline cellulose (Avicel^®^ PH 200, FMC Biopolymers, USA) as a filler, and magnesium stearate (Merch KGaA, Darmstadt, Germany) as a lubricant and antiadhesive agent. For preparation of dissolution media, KH_2_PO_4_ (Merch KGaA, Germany), NaOH (Merch KGaA, Germany), HCl 37% (Panreac Quimica S.A.U., Barcelona, Spain) and purified water (reverse osmosis, Faculty of Pharmacy, Ljubljana, Slovenia) were used.

### 2.2. Methods

#### 2.2.1. Preparation of Liquid SMEDDS Loaded with Carvedilol

Liquid SMEDDS was prepared according to our previously published research [3]. It contained 20% of oil phase (consisting of castor oil and Capmul^®^ MCM EP, in ratio 1:1) and emulsifying phase (consisting of Kolliphor^®^ RH 40 as nonionic surfactant and PEG 400 as cosolvent, in ratio 1:1). Crystalline carvedilol (100 mg of carvedilol per 1 g SMEDDS) was added to homogenous mixture of SMEDDS, heated to 50 °C and stirred until dissolved in SMEDDS (for approximately 3 h, with stirring speed of 60 rpm). The prepared liquid SMEDDS was transparent and yellowish in color.

#### 2.2.2. Granulation Dispersion Preparation

GD consisted of SMEDDS and water in a ratio of 70:30 with a variable addition of povidone K25 as a binder. The binder concentration differed in regard to the mesoporous carrier used, as each carrier required a specific binder amount to form optimal size granules with adequate properties. The composition of GD depending on the mesoporous carrier used is presented in Table 2 in Section 3.1 as well as the amount of each GD added, as it varied with carrier type, due to the difference in adsorption capacities between them.

#### 2.2.3. Determination of Granulation Dispersion Rheological Properties

The GD viscosity was evaluated using a rotational rheometer (Physica MCR 301, Anton Paar GmbH, Graz, Austria) with cylindrical measurement system (CC27/T200/SS). All measurements were performed at 25 °C temperature, with sample size of 20–25 mL. The shear rate during the rotational tests ranged from 1 to 100 s^−1^. The viscosity was compared between the samples as the value of read measurements at the lowest shear rate (1 s^−1^). Carvedilol-loaded SMEDDS dispersion (with SMEDDS:water ratio 70:30) was used as the reference sample, as it represents the system viscosity without povidone K25 added.

#### 2.2.4. SMEDDS Solidification by Manual Wet Granulation

In order to produce SMEDDS granules with optimal SMEDDS content, granulations were performed manually, in the paten with the pestle, using five mesoporous carriers (Fujicalin^®^ SG, Neusilin^®^ US2 and three silica-based: Syloid^®^ 244 FP, Syloid^®^ XDP 3050 and Aeroperl^®^ 300), before scaling up to the high-shear granulator. Formulations needed to be adjusted for each carrier and differed in the amount of GD added, as well as the amount of binder needed to form granules. Firstly, the mesoporous carrier was weighted into the paten and the GD was added dropwise, while continually mixing with the pestle. The granulation process took approximately 20 to 30 min, depending on the amount of GD added. The granulation endpoint was evaluated visually, regarding the size of the granules formed, as well as the granules touched by fingertips. Afterwards, the mass was stirred for two more minutes in order to distribute the granulation liquid between the particles as thoroughly as possible. The wet mass then was further sifted through a sieve with mesh size of 1000 µm and dried on a laboratory tray dryer at 70 °C, until a moisture content 2–3% was achieved (approximately 20–25 min).

#### 2.2.5. SMEDDS Solidification by High-Shear Wet Granulation

Solid SMEDDS granules were produced using HS granulator Pro Cept 4M8-Trix. The carrier was weighed in an amount to fill about a third of the volume of the glass one-liter bowl. Process parameters were set as shown in Table 1. Depending on mesoporous carrier used, the GD was sprayed or dripped onto the carrier inside the bowl. The end point of the granulation was determined based on the amount of liquid added (in reference to previous manual granulation), the visual appearance regarding the size of the granules formed and the granules feel under fingertips. Upon reaching endpoint, the granulate was kneaded for an additional 2 min to distribute the liquid evenly among the particles (using the same conditions). The processed mass was further sieved and dried in tray dryer by the same procedure as the manual granulate (Section 2.2.4). In comparison with manual granulation, the drying time was prolonged (45–55 min), due to the increased amount of material used in the process.

#### 2.2.6. Loss on Drying

SMEDDS granules loss on drying was measured on the thermogravimetric analytical balance (BÜCHI Moisture Analyzer B-302). A total of 2–3 g of the granules was placed on the aluminum pan to cover the pan surface in a thin layer. The measurement conditions were set on 85 °C for 15 min. The result was displayed as the percentage that represents the proportion of moisture in the granules, as the moisture content analysis is based on sample weight reduction due to water evaporation.

#### 2.2.7. Granules Size and Size Distribution Measurement

The size and size distribution of the produced SMEDDS granules as well as the mesoporous carriers particles were measured using the Mastersizer 3000 device by placing approximately 1 g of the sample into an Aero S dispersion cell. Measurement conditions were 1.5 bar of air pressure with 20% feed rate. Mastersizer 3000 uses laser diffraction to obtain the results, which are presented as d_10_, d_50_ and d_90_ values (particle size in μm) as well as SPAN (particle size distribution).

#### 2.2.8. Evaluation of Granules Flow Properties and Compressibility

SMEDDS granule’s flow properties were evaluated according to Ph. Eur. 10th (2.9.36. Powder flow) [23]. For the flow time measurement, about 15 g of the sample was poured into a standard glass funnel with a neck diameter of 10 mm, and the time needed for the entire sample to flow out was calculated per 100 g of sample and expressed as the flow time in s/100 g. The angle of repose measurement was performed on the formed powder heap using a ruler. All measurements (for flow time and angle of repose) were performed about 10 times and expressed as average values.

For the evaluation of the granules’ Carr index properties, about 70–80 g of granules was accurately weighted and gently placed into a 100 mL plastic cylinder. The volume of the sample was measured as the bulk volume. Further, the cylinder was tapped 1250 times with a tap density tester (VanKel 50–1100) to determine the tapped volume. All measurements were performed in triplicate and expressed as average values. Bulk and tapped volumes were used for the calculation of bulk and tapped densities, and Carr index as an indicator of the produced granules flow.

#### 2.2.9. Determination of Carvedilol Content

The carvedilol content in SMEDDS granules and tablets was determined using UV spectroscopy, by measuring the absorbance at 284 nm wavelength (as already described by Mandić J. et al. [3]). Precisely weighed samples with theoretical content of 12.5 mg API were quantitatively transferred into a 500 mL measuring flask and partially filled with a medium (diluted HCl solution with pH = 1.2). Methanol was used as a cosolvent, as it does not absorb UV light at the specified wavelength. Thereafter, the flask was sonicated for 30 min, following by 30 min stirring on 50 °C, and an additional 30 min of sonication. For the final sample preparation, the flask was filled up with the medium to the volume mark.

Then, 10 mL of the prepared sample was filtered into a cuvette through a 0.45 μm RC membrane filter. The absorbance of the sample was measured at the determined wavelength, in reference to the medium used as a blank. The concentration of carvedilol in the sample was calculated based on the previously determined carvedilol calibration curve (2.5 to 30 μg/mL; R^2^ = 0.99995), which was further calculated to express the carvedilol content (mg) per g of solid granules.

#### 2.2.10. Granules Surface Morphology

The surface morphology of SMEDDS granules was observed under 150–1000× magnification, using a Supra 35VP scanning electron microscope (SEM) at 1 kV accelerating voltage. The prepared sample consisted of granules carefully put onto carbon tape, previously glued to a metal plate used as a carrier.

#### 2.2.11. Assessment of Carvedilol Physical State

The physical state of carvedilol in the selected SMEDDS granules was assessed by differential scanning calorimetry (DSC) and X-ray diffraction (XRD) analysis. In addition to the produced granules, crystalline carvedilol and mesoporous carriers were also evaluated. 

By differential scanning calorimeter (DSC1 STARe System, Mettler Toledo), the samples (3–7 mg) were heated in an aluminum pan with perforated lid, from 0 °C to 160 °C with a scanning rate of 10 °C/min and nitrogen gas flow of 50 mL/min. As a reference, an empty aluminum pan was used. Finally, the output data were evaluated by STARe V9.30 software program (Mettler Toledo, Columbus, OH, USA).

The XRD diffractograms were obtained by PANalytical PW3040/60 X’Pert PRO diffractometer (CuKα1 radiation, λ = 1.5406 Å) using the continuous scanning mode in the 2θ range from 3 to 32° and the step of 0.033° per 250 s.

#### 2.2.12. Compaction into SMEDDS Tablets

Tablets were prepared from HS granules using a single-punch tablet press (Kilian SP 300), in addition to other excipients needed to enable tableting. SMEDDS tablets were compressed using a 12 mm flat face round punch with each tablet containing approximately 12.5 mg of carvedilol.

The tableting mixture consisted of 25% *m*/*m* of SMEDDS granules (amount containing 12.5 mg of carvedilol), 5% *m*/*m* of copovidone (Kollidon^®^ VA64), 5% *m*/*m* of sodium croscarmellose (Ac-Di-Sol), 1% *m*/*m* of magnesium stearate and 64% *m*/*m* of microcrystalline cellulose (Avicel^®^ PH-200).

#### 2.2.13. Evaluation of SMEDDS Tablets

All SMEDDS tablets were evaluated for hardness (VanKel 200 Tablet Hardness Tester), disintegration time (Erweka ZT4) and friability (Erweka TAR 10), according to the criteria in corresponding monographs from Ph. Eur. (2.9.1. Disintegration of tablets and capsules; 2.9.7. Friability of uncoated tablets; 2.9.8. Resistance to crushing of tablets) [23].

Tablet hardness was determined for 10 tablets, and the value was presented as the average.

The tablet disintegration time was measured according to Ph. Eur. Only three tablets were tested, due to lack of samples. The time needed for the tablet to disintegrate, from the moment the basket is placed in the water, was marked as disintegration time.

Friability testing (Ph. Eur. 10th 2.9.7. Friability of uncoated tablets) was performed with 10 tablets. Weighed tablets were placed in a drum, which rotated for 4 min at 25 rpm. After the experiment, SMEDDS tablets were weighed again, and the friability was calculated according to Equation (1):

(1) $$\mathrm{friability}\;\left(\%\right)\;=\;\frac{m\left(before\;test\right)-\;m\left(after\;test\right)\;}{m\left(before\;test\right)}\times 100\%$$

#### 2.2.14. Evaluation of Self-Microemulsifying Properties 

To assess self-microemulsifying properties, about 100 mg of SMEDDS granules were dispersed in 25 mL of medium (210 mL in the case of SMEDDS tablets) and stirred on a magnetic stirrer (200 rpm) at room temperature, for 30 min to ensure complete redispersion [2]. Three different media were used: purified water, diluted HCl solution with pH 1.2 and phosphate buffer with pH 6.8. Upon mixing, dispersions were left to stand for about 20 min, to allow undissolved carrier remains to settle down. The supernatants were then filtered through a 0.45 μm RC membrane filter into a cuvette. Droplet size and polydispersity index (PDI) of filtered dispersions were determined at 25 °C using photon correlation spectroscopy (PCS) with Zetasizer Ultra. Each sample was measured in triplicate, and the results were presented as average values.

#### 2.2.15. In Vitro Dissolution Testing

Dissolution studies of in vitro carvedilol release from SMEDDS granules and tablets were conducted using Ph. Eur. Apparatus 2 dissolution tester with rotating paddles (VanKel VK 7010; Ph. Eur. 10th 2.9.3. Dissolution test for solid dosage forms [23]). 

Samples of SMEDDS granules and tablets (containing 12.5 mg of carvedilol) were tested in triplicate, and the result presented as an average value with corresponding standard deviation. They were placed into a 900 mL dissolution vessel filled with selected medium (diluted HCl solution with pH = 1.2 or phosphate buffer with pH = 6.8) and heated to 37 ± 0.5 °C in paddles rotating at 50 rpm. As a reference samples, 12.5 mg of pure crystalline carvedilol and adequate mass of liquid SMEDDS were used. At predetermined time intervals (5, 10, 20, 30, 45, 60, and 120 min, and additional sampling point time point at 240 min for pH 6,8 media) 10 mL of sample was withdrawn and filtered through a 0.45 μm pore RC membrane filter. The withdrawn medium was replaced with the same volume of fresh/pure medium, to maintain 900 mL in total volume in the vessel. At the end of testing period, the paddles rotation speed was increased to maximum 250 rpm for an additional 5 min to ensure the complete release of the API, whereupon the final sampling was conducted.

Samples were further analyzed using UV spectroscopy, and the absorbance was measured at 284 nm wavelength. The carvedilol concentration was determined in relation to the calibration curves obtained in both media; the in vitro release profile was plotted as the cumulative percentage of released carvedilol versus time.

## 3. Results and Discussion

### 3.1. Formulation Development and Characterization of SMEDDS Granules

#### 3.1.1. Manual Wet Granulation with SMEDDS-Based Granulation Dispersion

Five different mesoporous carriers (Fujicalin^®^ SG, Neusilin^®^ US2 and three silica-based ones, Syloid^®^ 244 FP, Syloid^®^ XDP 3050 and Aeroperl^®^ 300), previously shown from the perspective of SMEDDS solidification [2,3,9,10,24,25,26], were chosen for the development of the formulation by manual wet granulation, aiming to identify products suitable for scaling up by HS granulation. They differ in chemical composition, porosity and oil adsorption capacity, which is 3.00 g/g for Syloid^®^ 244FP [27], 3.20 mL/g for Neusilin^®^ US2 [28], 1.10 mL/g for Fujicalin^®^ SG [29], 2.74 g/g for Syloid^®^ 3050 XDP [30] and 2.75 g/g for Aeroperl^®^ 300 [30], based on the literature data. The characteristics of the produced granules were then compared with regard to the used solid carrier and method of granulation (manual or HS granulator).

For each carrier, the optimal amount of GD and the binder needed to form granules suitable in size and flow properties, was determined. The aim of formulation adjustments was to optimize SMEDDS incorporation into the production of free-flowing granules, while maximizing the liquid load, as high SMEDDS content relates to high carvedilol content and, consequently, a smaller final dosage form at the same time.

Firstly, the maximum amount of added liquid was determined, as carriers differ in porosity and consequently in liquid adsorption capacity. Secondly, the amount of binder in GD was varied until obtaining granules with adequate flowability. Binder concentration in GD was than varied in the following ranges based on the carrier dependent characteristics of formulation: 2–18% for Syloid^®^ 244FP, 6–20% for Neusilin^®^ US2, 2–10% for Fujicalin^®^ SG, 5–30% for Syloid^®^ 3050 XDP and 10–20% for Aeroperl^®^ 300 (Table S1). With lower binder amounts, the granules were more likely to be greasy to the touch and showing very poor flow properties. By increasing the binder concentration in the GD, the carvedilol content in granules decreases; nevertheless, it allows us to produce granules with good flowability while achieving an adequate liquid load. The composition and characteristics of most optimal formulations are presented in Table 2.

The optimal binder concentration in GD varied greatly between products obtained by different carriers (i.e., from 5% for Fujicalin^®^ SG to 25% for Syloid^®^ XDP 3050). Therefore, the amount of added liquid (describing the mass of microemulsion composed of 70% SMEDDS and 30% water added within GD) was considered a more appropriate parameter for describing the carrier’s adsorption capacity in comparison to the amount of added GD, which additionally includes different share of binder as well.

By optimizing the share of binder in GD, it was possible to produce granules with up to 66% of SMEDDS. As expected, more liquid could be loaded into mesoporous carriers with higher adsorption capacities, as reported in the literature data. Neusilin^®^ US2 and Syloid^®^ 244FP thus proved to be the most efficient carriers, as they adsorbed 3.03 g and 2.68 g of liquid per 1 g of the carrier, respectively, while maintaining the characteristics of free-flowing granules after drying. This is a substantial improvement, as SMEDDS represented almost 2/3 of final granules weight. Consistent with specific surface area values (40 m^2^/g vs. 300 m^2^/g), the lowest SMEDDS loading was obtained for Fujicalin^®^ SG that was able to absorb not more than 0.88 g of per 1 g of the carrier; an increase in GD addition resulted in sticky granules with very poor flow properties.

The outlier was Syloid^®^ XDP 3050, with liquid incorporation of 1.93 g per 1 g of carrier, which is considered low, bearing in mind that its declared oil adsorption capacity was relatively high. This is in line with our previous study on the solidification of SMEDDS by the spray drying technique, where the performance of Syloid^®^ XDP 3050 was inferior to Syloid^®^ 244FP and Neusilin^®^ US2 as well [4]. In the present study, optimal content of povidone K25 in GD is rather high (25%), and it is thus possible that binder particles remained partially suspended in GD, clogging the pores on the carrier surface and hindering SMEDDS penetration further into the particles. In keeping with this, Syloid^®^ XDP 3050 based granules exhibited worse flow characteristics (Table 3), most probably related to SMEDDS dispersion mainly adsorbed onto the particle surface, leading to a stickier granules surface that further obstructed granule flow.

According to the results of SMEDDS granules characterization presented in Table 3, all manually produced SMEDDS granules exhibited excellent flow properties with regard to the angle of the repose values. However, the flow time turned out to be better classifier, with two silica-based SMEDDS granules (G_m_ SYL_244_ and G_m_ AER) exhibiting flow time below 10 s/100 g. Nevertheless, it was below 14 s for all other granules, so still no distinguishable difference between the five mesoporous carriers could be made. On the contrary, the influence of carrier type was better pronounced when considering the Carr index, as SMEDDS granules could be grouped into three different categories (according to Ph. Eur. criteria). Accordingly, G_m_ SYL_244_ and G_m_ FUJ were classified into the group with fair flow properties, G_m_ NEU and G_m_ AER showed passable flowability, while G_m_ SYL_3050_ exhibited very poor flow properties. Despite G_m_ FUJ exhibiting fair flowability, Fujicalin^®^ SG was omitted from further studies, due to the poor SMEDDS loading efficiency. Additionally, the results obtained for G_m_ AER were somehow discouraging, when considering the study of Aeroperl^®^ 300-based particles prepared by the adsorption method, both exhibiting significantly better flow properties than Neusilin^®^ US2 [24].

As at this stage of investigation, the aim was to select a suitable formulation for scaling up to the HS granulator, so the viscosity of GD was considered one of the critical factors to allow unobstructed addition to the granulation vessel and spraying through the nozzle. Ideally, it should be kept low, which enables appropriate particle agglomeration [3,31,32]. Therefore, the viscosity of each GD was measured also before manual granulation process, as they differ in binder concentration. As expected, the viscosity of GD was dependent on the amount of povidone K25 added (Figure S1), with the lowest viscosity of 0.62–0.67 Pa·s measured for GD with the lowest binder concentration and vice versa (Table S2).

Regarding that, among GDs used for manual granulation, those adjusted for Aeroperl^®^ 300 and Syloid^®^ 3050 XDP have significantly higher viscosities, which most probably contributed to poor SMEDDS loading into Syloid^®^ 3050 XDP particles. This is in accordance with a study on liquisolid systems preparation, where low liquid viscosity was correlated to fast liquid loading into the pores of the carrier [33].

#### 3.1.2. High-Shear Granulation with SMEDDS-Based Granulation Dispersion

Based on SMEDDS loading and flow characteristics, it was decided to scale up the study in the direction of HS granulation with Neusilin^®^ US2 and Syloid^®^ 244FP, as the most promising two results. The low viscosity of GD with 7% of povidone K25, which was chosen as optimal for the granulation of Syloid 244 and Neusilin US2, supported their selection also from this point of view.

When producing SMEDDS granules by HS granulation, a slightly lower amount of GD was added to the solid carriers (2.27 g to Neusilin^®^ US2 and 2.33 g to Syloid^®^ 244FP) as compared to their manually produces counterparts (3.27 g to Neusilin^®^ US2 and 2.87 g to Syloid^®^ 244FP). In addition to being cautious not to exceed the liquid adsorption capacity of the carriers, this was most probably related to faster GD addition, allowing the liquid less time to penetrate into the pores compared to manual granulation. Nevertheless, the SMEDDDS content in granules was still high (60% *m*/*m*). Moreover, the process was much more efficient, as the amount of processed material increased sevenfold in shorter granulation time. Considering also the good flow properties (Table 3), essential for formulation design when further tableting or filling into capsules is required, the HS granulation technique has proved to be not only a promising SMEDDS granulation method, but superior in comparison to other solidification techniques, such as fluid-bed granulation or spray drying, where up to 45% or 56% of SMEDDS uptake was obtained, using Neusilin^®^ US2 as a carrier, respectively [2].

Slightly broader SPAN values (1.85 vs. 1.22) and a lower d_50_ value (also presented in Table 3) showed that some material was left ungranulated, probably due to the lower amount of liquid added. For G_hs_ NEU, this is also evident from the graphical representation of particle size distribution by volume (Figure S2). Unexpectedly, scaling up of the production process resulted in a 3% lower process yield in the case of G_hs_ NEU, although smaller sized particles could pass through the sieve more easily. However, more material was lost in the HS vessel, as HS forces obtained during granulation would push the material to the wall of granulation vessel, contributing to its sticking and not being able to empty it completely. Prolonging the granulation time by reducing the flow rate of GD may contribute to better granulation efficacy, shown as increased process yield and SMEDDS content. In that manner, SMEDDS would be incorporated deeper in pores, with such material probably being less sticky on the chamber wall.

Unlike for G_hs_ NEU, the process yield increased by 4% for G_hs_ SYL_244_ compared to manually produced granules. The reason is likely to be granulation in a closed chamber, as Syloid^®^ 244FP is a very voluminous, lightweight material with a low bulk density (0.06 g/mL [34]). When granulating manually, there was some loss during the process, in comparison to the closed system of HS granulation that constantly kept the material inside the chamber. Another difference in the process lays in the manner of liquid addition. Specifically, the GD was added dropwise onto Syloid^®^ 244FP rather than being sprayed as in the case of Neusilin^®^ US2. Due to the Syloid^®^ 244FP small particles (3.5 µm) and low bulk density, GD could not be sprayed onto the carrier, as the filter of the HS granulator would clog when increasing air pressure through the nozzle. G_hs_ SYL_244_ were slightly smaller in size than the ones made manually (d_50_ values: 366 µm in comparison to 488 µm), the width of the distribution being approximately the same, and the whole amount of starting material was granulated (no overlapping with the curve corresponding to primary carrier particles, as seen on Figure S2).

Concerning flow properties, granules produced in the HS granulator showed enhanced flowability. G_hs_ NEU thus belongs to a fair grade, while its manually prepared counterpart classifies as being passable, according to Ph. Eur. criteria for the Carr index. G_hs_ SYL_244_ are still in fair grade, although on the upper limit toward good flowability. The flow time improved as well: for G_hs_ NEU, from 12.5 s per 100 g of granules to less than 10 s, while for G_hs_ SYL_244_ the value dropped from 8.8 per 100 g of granules to under 6 s, both in favor of HS granulation. This is likely due to higher shear forces used during the granulation process, resulting in more spherical shape granules, with smoother surfaces and less sharp edges that would inhibit the flow. This is in line with the SEM analysis (Figure 3), confirming the rounder shape of G_hs_ NEU granules in comparison to slightly irregular ones, prepared by manual granulation. 

To sum up, granules produced in HS granulator have a more homogenous particle shape (as seen in SEM images) and are slightly smaller in size (d_50_ value of ones with Neusilin^®^ US2 is 329 µm, while with Syloid^®^ 244FP it is 366 µm), both being preferable from the industrial perspective. In comparison to the manually produced granules SMEDDS loading was slightly lower, with G_hs_ NEU and G_hs_ SYL_244_ loading of 60% and 59% (respectfully) relative to granules weight. Nevertheless, these results represent a remarkable improvement of the solid SMEDDS flow properties, when taking into account the influence of high SMEDDS loading on the deterioration of the product’s flowability observed previously [2,35]. In comparison to other SMEDDS solidification methods that also utilized Neusilin^®^ US2 as a carrier, the difference was obvious: the product obtained by fluid-bed granulation exhibited passable-to-poor flow properties (Carr index value was 23–29%), with average flow time 25–30 s/100 g, while the product prepared by the direct adsorption method exhibited only fair flow properties (Carr index value was 18%, angle of repose 38°) [11]. The superior behavior of SMEDDS HS granules was in some respects expected, due to the differences in the granule formation and growth during the process itself and, consequently, the production of more regularly shaped and spherical SMEDDS granules using the HS granulator [36,37].

#### 3.1.3. The Impact of Carrier Type and Granulation Method on SMEDDS Granules Surface Morphology

**Figure 1:**
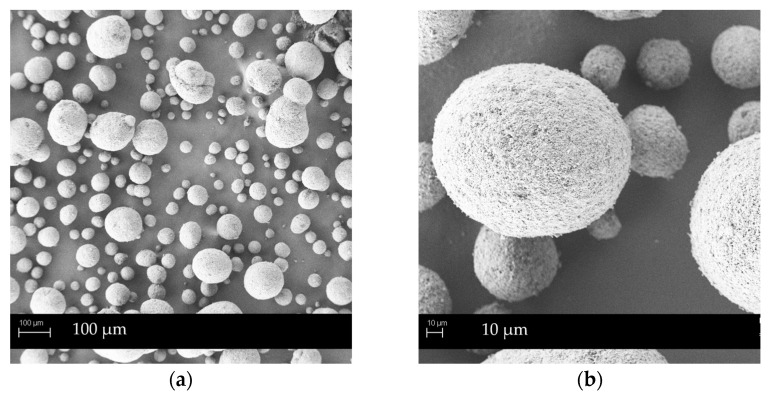
SEM images of Neusilin^®^ US2 particles: (**a**) under magnification 200×; (**b**) under magnification 1000×.

**Figure 2:**
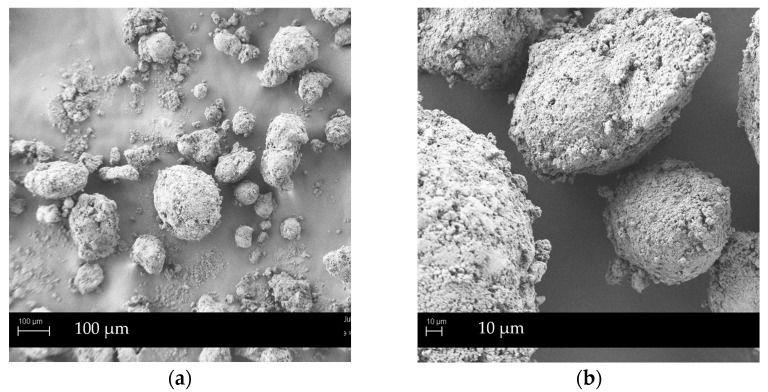
SEM images of G_m_ NEU: (**a**) under magnification 200×; (**b**) under magnification 1000×.

**Figure 3:**
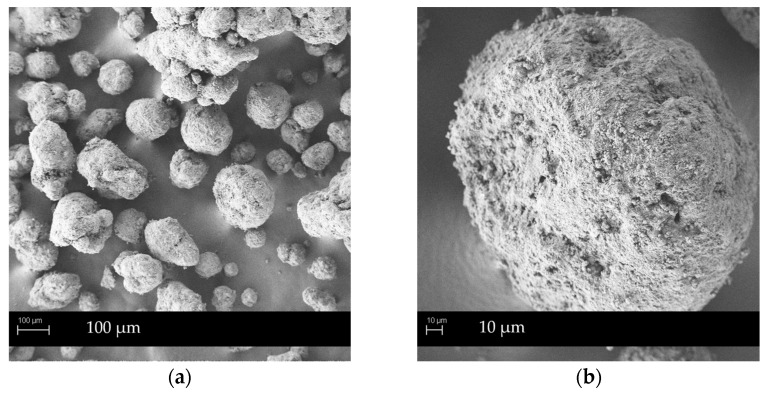
SEM images of G_hs_ NEU: (**a**) under magnification 200×; (**b**) under magnification 1000×.

SMEDDS granules surface morphology was evaluated by SEM analysis to investigate the influence of different formulation and SMEDDS granulation methods on the particles shape, surface and pores, filled with the lipid-based dispersion. Figure 1, Figure 2 and Figure 3 show SEM images of G_m_ NEU, G_hs_ NEU as well as initial mesoporous Neusilin^®^ US2 particles, while SEM images of other SMEDDS granules (G_m_ SYL_244_, G_hs_ SYL_244_, G_m_ FUJ, G_m_ AER and G_m_ SYL_3050_) are presented in Figures S3–S7.

Generally, G_m_ NEU granules look spherical in shape. At a lower magnification, a substantial amount of crushed material can be seen, indicating that, during manual granulation, round spheres of primary Neusilin^®^ US2 particles were crushed with the pestle [35]. These fragments subsequently glued to the granules’ surface, giving them slightly irregular shape that could degrade the flow properties. On the contrary, granules prepared using high-shear forces maintained the rounder shape of the primary particles better and therefore exhibited better flow properties (also confirmed by flow time and Carr index values; Table 3).

SEM images show G_m_ SYL_244_ with a lot of fragmented or even ungranulated primary particles, of sizes below 100 µm. However, most of the particles look spherical in shape, contributing to good flow properties of the corresponding granules. When prepared by the HS granulator, more distinct granules with a rough surface, possibly related to unfilled pores of the carrier, are visible. This allows us to presume that G_hs_ SYL_244_ with a higher SMEDDS load and correspondingly smoother surface could exhibit more uniform size and even better flow properties. Regarding the particle size, SEM analysis showed that the granule size is not in correlation with the d_50_ value determined by the laser diffraction method, as the latter proposes that granules are completely spherical in shape, which was disproved by SEM observation as well. Inadequate sample preparation (described in Section 2.2.10) probably led to such a result, as some of larger particles fell off from the metal plate, which was used as a carrier.

During granulation with Fujicalin^®^ SG, some granules were crushed into smaller pieces, probably due to pestle pressing, resulting in the irregular shape of G_m_ FUJ. Similarly, a lot of fragmented pieces stuck to the surface of G_m_ SYL_3050_ and (to the lesser extent) G_m_ AER, probably leaving the surface difficult to wet with the granulation liquid and letting pores of carrier be unfilled, which further resulted in the poor flow properties.

Ultimately, the differences in the SMEDDS granules shape and surface, depending on the granulation method, were found to be in accordance with the literature data, where HS granulation enabled the formation of more spherical particles with more uniform size distribution. Due to the higher shear forces obtained in the granulator, the filling of the carrier’s pores was more even, resulting in a smoother granules surface and less sharp edges that further reflected in the improvement of the flow properties [37,38].

#### 3.1.4. Carvedilol Physical State in SMEDDS Granules

**Figure 4:**
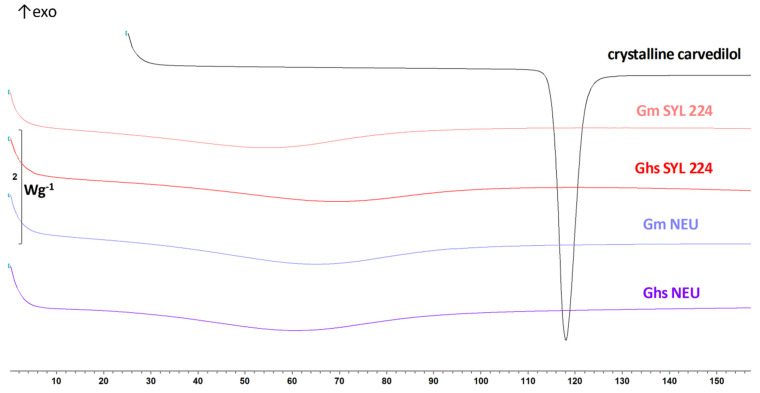
DSC curves, representing pure crystalline carvedilol and produced granules G_m_ SYL_224_ G_hs_ SYL_224_, G_m_ NEU and G_hs_ NEU.

DSC analysis was performed to characterize the carvedilol physical state in solid SMEDDS. When incorporated in SMEDDS formulation, carvedilol was in a dissolved state. However, for the GD preparation purpose, SMEDDS was diluted with water (in ratio 70:30) which potentially increased the possibility of carvedilol precipitation [2,3,39]. The DSC results showed no thermal events visible on the thermograms of the analyzed granules (Figure 4). In accordance to the previously published research [2], the carvedilol melting temperature did not significantly change in physical mixtures with corresponding carriers (1:1), as its onset was determined at 115.09 °C. Most importantly, none of SMEDDS granules’ DSC curves showed the melting peak of crystalline carvedilol. This allow us to presume that carvedilol is either in an amorphous or molecularly dispersed state.

**Figure 5:**
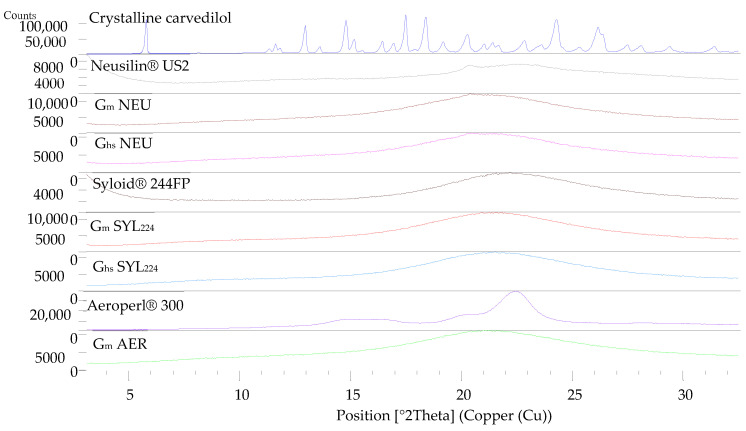
XRD diffractograms of pure crystalline carvedilol, three pure mesoporous carriers (Neusilin^®^ US2, Syloid^®^ 244FP and Aeroperl^®^ 300) and corresponding granules (G_m_ NEU, G_hs_ NEU, G_m_ SYL_224_ G_hs_ SYL_224_, G_m_ AER and G_hs_ AER).

The possible presence of crystalline carvedilol was further investigated in granules G_m_ NEU, G_hs_ NEU, G_m_ SYL_224_ G_hs_ SYL_224_, G_m_ AER and G_hs_ SYL_3050_ using XRD analysis, with pure crystalline carvedilol and corresponding pure carriers used as reference samples (Figure 5).

Many diffraction peaks were seen on pure carvedilol diffractogram, from 5 to 30° of 2-theta value. All carriers were in an amorphous state, considering smooth shape of XRD curves, without pronounced diffraction peaks. In granules, there were no crystalline diffraction lines visible either, therefore carvedilol’s amorphous state in granules was confirmed by XRD analysis. 

Overall, the results of DSC and XRD analysis indicate that wet granulation, used as SMEDDS solidification technology, did not initiate or cause carvedilol recrystallization, as it was present in either an amorphous state and/or remained dissolved within SMEDDS.

### 3.2. Formulation Development and Characterization of SMEDDS Orodispersable Tablets

In final phase of this research, the goal was to produce self-microemulsifying tablets with high carvedilol content and adequate quality attributes—foremost adequate mechanical properties (tablet hardness, low friability, fast disintegration time) in addition to a fast in vitro release profile.

As the SMEDDS granules’ flow properties improved with HS granulation, G_hs_ SYL_224_ and G_hs_ NEU were chosen for further compressing into tablets, with the addition of appropriate excipients, required for the process. The formulation development and corresponding tableting mixture compositions are presented in Table 4.

Firstly, pure granules were compressed using a single punch laboratory tablet machine, without any added excipients (TM_0_). The produced tablets were greasy to the touch, as high compression forces probably caused SMEDDS leakage from the carrier pores [2,11,35]. The high SMEDDS load in tablets was also reflected in the low hardness of ≈24 N for Neusilin^®^ US2 24 and ≈ 13 N for Syloid^®^ 244FP granules, which was also reported before by other authors [26]. Tablet hardness was constantly low, despite the increase in the compression force. Thus, it was necessary to reduce the share of SMEDDS granules and add other excipients to ensure a suitable tablet hardness (>100 N). Therefore, tableting mixture TM_1_ containing 50% *w*/*w* of SMEDDS granules, 5% *w*/*w* of the binder (Kollidon^®^ VA64), 5% *w*/*w* of disintegrant (Ac-Di-Sol^®^), 1% *w*/*w* Mg-stearate and the rest of filler (microcrystalline cellulose; Avicel^®^ PH-200) was developed. The latter is well established as direct compression enhancer that simultaneously adsorbs the leaked SMEDDS from compressed granules while maintaining good compaction properties, both of which favor SMEDDS tablet production [2,40,41].

Accordingly, with increasing the compaction properties of the mixture, the tablet hardness increased, too. The resulting tablet hardness of ≈34 N was, in any case, a slight improvement in comparison to SMEDDS tablets produced in the study of Mandic et al., in which the tablets with similar share of SMEDDS granules (43.2% *w*/*w*) exhibited hardness of ≈29 N [2]. Nevertheless, this result was unsatisfactory, so in the next tableting mixures, the binder share was raised to 10% *w*/*w* (TM_2_) or the disintegrant was reduced to 3% (TM_3_). Surprisingly, there was no desired improvement in tablet hardness.

A remarkable progress in tablet hardness was finally achieved when the SMEDDS granules share was reduced to 25% *w*/*w*. The final tableting mixture TM_4_ also contained 5% *w*/*w* of Kollidon^®^ VA64 and Ac-Di-Sol^®^. 

Eventually, SMEDDS tablets with ≈122 N hardness were successfully produced, under a compression force of 9.5 kN using G_hs_ NEU granules. By applying the same compression force to TM_4_ containing G_hs_ SYL_244_, the result was approximately 95 N. Interestingly, to obtain the same hardness of tablets with G_hs_ SYL_244_ (T-SYL_244_), as for tablets with G_hs_ NEU (T-NEU), the compression force needed to be increased to almost 11 kN, demonstrating the influence of carrier type on the hardness of the SMEDDS tablets. A possible explanation could lay in the formation of a stronger interparticle bonding between magnesium aluminometasilicates particles in G_hs_ NEU, then between amorphous silica particles in G_hs_ SYL_244_ (as the only difference is in mesoporous carrier type).

With the purpose of achieving suitable mechanical properties of SMEDDS tablets, the major drawback was the final tablet mass. As the tablet mass eventually depended on single dose of carvedilol (12.5 mg), the corresponding amount of granules needed to be kept constant, while the amounts of additional excipients were proportionally increasing. Hence, to maintain the adequate dose of carvedilol in single SMEDDS tablet, it finally weighed ≈ 845 mg.

After SMEDDS tablet formulation optimization, the final tablets mechanical properties were evaluated by corresponding to Ph. Eur [23]. All friability results met the Ph. Eur. criteria, namely friability of 0.12% for Neusilin^®^ US2 and 0.22% in case of Syloid^®^ 244FP used as carriers, which is most likely associated with the higher tablet hardness of T-NEU. The disintegration test was performed with a reduced number of tablets (due to the small batch size), with three instead of six tablets. For both mesoporous carriers, the average disintegration time met the criteria, as it was remarkably shorter than the required 15 min (29 s for T-NEU and 51 s for T-SYL_244_). Therefore, such tablets actually met the criteria (disintegration time shorter than 3 min) for orodispersible tablets as well [42].

### 3.3. Improvement of Carvedilol In Vitro Dissolution Properties and Self-Microemulsifying Properties of Solid SMEDDS

Self-microemulsifying properties, or the ability to form microemulsion upon dispersion and keep the drug molecules dissolved inside these droplets, are important due to the preservation of the initial advantage of formulation as SMEDDS. The solidification process can influence microemulsion formation after redispersion in terms of the droplet size increase [2,11,43].

All prepared dispersions of SMEDDS samples were optically transparent after filtration, indicating the presence of colloidal sized droplets, as also confirmed by PCS (z-average under 100 nm). The obtained results are shown in Table 5.

Upon redispersion of granules, the average droplet size (z-average diameter) was <82 nm in all three media (distilled water, media with pH 1.2 and pH 6.8); therefore all measured dispersions can be classified as microemulsions [1,44]. In the water dispersion of liquid SMEDDS without carvedilol, the determined droplet size diameter was around 23 nm (peak 1), with PDI slightly above zero (0.07), pointing out the monodisperse system. On the other side, at the corresponding dispersion with the model drug incorporated, the additional intensity peak (peak 2) was detected at about 150 nm, probably due to API, that partially precipitated in a form of nanosuspension [2,3]. Peak 2 with values > 1000 nm could indicate the presence of microparticles of impurities, as the intensity response was registered also in liquid SMEDDS without API. In the case of solid SMEDDS, it is also possible that the referred peak is present due to the remains of fine, nanometer-size carrier particles despite filtering the samples before measurements [45]. 

By observing liquid SMEDDS peaks that correspond to the microemulsion (peak 1, in water medium), there is no tangible difference in droplet size in comparison to the results obtained from granules or tablets. Concerning media with pH 1.2 and 6.8, the transformation to solids also did not affect the droplet size. G_m_ NEU stood out with microemulsion within the narrowest droplet size range formed, as the z-average values were 21–26 nm, with PDI from 0.17 to 0.24 (in all three media).

During SMEDDS solidification based on Neusilin^®^ US2, no change in the droplet size of resulting dispersions was observed, regardless of the wet granulation method. In support of this outcome, the microemulsion formation also was not affected by the solidification process, as reported by Cho et al. In the study of sirolimus-loaded SMEDDS granules, the droplet size upon redispersion was not changed in comparison to liquid SMEDDS [46]. The average droplet size did not increase due to SMEDDS solidification in SMEDDS produced by fluid bed granulation (<30 nm [3]) or with adsorption on solid carriers (<80 nm [35]), which are contrary results compared with earlier studies using other SMEDDS solidification techniques [11].

Moreover, previous studies showed that tablet compaction and the addition of water-insoluble excipients, such as mesoporous carriers, can impair self-microemulsifying properties [10,18]. However, in our study, the average droplet size increased just slightly after the compaction, remaining in the domain of microemulsion droplet size. For T-NEU, the average droplet sizes were ≈ 78 nm, 36 nm and 45 nm (in water medium with pH 1.2 and pH 6.8, respectively) in comparison to G_hs_ NEU with 23 nm, 22 nm and 25 nm. When Syloid^®^ 244 FP was used as carrier, the difference in droplet size between T-SYL_244_ and G_hs_ SYL_244_ was somewhat bigger than with Neusilin^®^ US2. The widest droplet size distribution was determined for SMEDDS tablets, in comparison to SMEDDS granules redispersion, as supported by increased PDI values, which is also consistent with the literature [2].

Ultimately, the formation of microemulsion was confirmed upon redispersion of all granules and tablets. The difference between solid SMEDDS could be found in the z-average value upon tableting, with formation of droplets larger than 50 nm, although this was still within the microemulsion range (<80 nm). Therefore, the self-microemulsifying ability of solid SMEDDS was preserved, which is in favor of the initial formulation approach for improving the gastrointestinal absorption and bioavailability of carvedilol as a poorly water-soluble drug.

**Figure 6:**
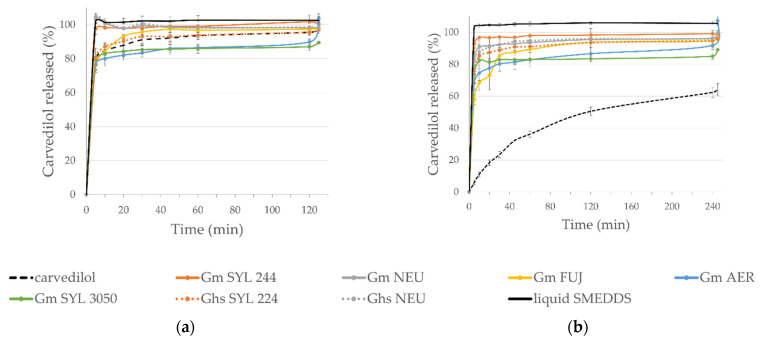
In vitro carvedilol release profiles in dissolution media with pH: (**a**) 1.2; (**b**) 6.8; for all SMEDDS granules in reference to crystalline drug and liquid SMEDDS.

Hence, with the aim to confirm that SMEDDS and its consequent solidification is suitable formulation approach for carvedilol, in vitro dissolution studies were conducted (Figure 6). Since it is already dissolved in liquid SMEDDS, carvedilol should be readily available for absorption in gastrointestinal tract. Carvedilol exhibits the properties of weak base due to amine group in the structure, so it was expected to dissolve more slowly in a test medium with pH 6.8 in comparison to the pH 1.2 medium [47]. A comparison of the dissolution profiles of SMEDDS granules, five of them prepared manually and an additional two produced using the HS granulator, is shown in Figure 6.

As seen from the figure, liquid SMEDDS exhibited not only faster, but complete release in both media within the first 5 min in comparison to crystalline carvedilol (≈80% in pH 1.2 and ≈6% in pH 6.8, with maximum of 64% of released drug in the latter), which confirmed that the incorporation of carvedilol in SMEDDS improved its dissolution, an essential prerequisite for good bioavailability. Furthermore, this advantage aimed to be preserved with transformation into solid SMEDDS. With dissolution testing in pH 1.2 medium, all SMEDDS granules exhibited complete carvedilol release, as the percentage of the released drug was >97%, except in the case of G_m_ SYL_3050_, where it was 90%. However, the results obtained for SMEDDS granules differ regarding the mesoporous carrier used. While G_m_ SYL_244_ and G_m_ NEU showed complete drug release in 5 min, formulations with other carriers exhibited a slower and similar release rate to crystalline carvedilol. 

In addition to being a discriminatory medium for carvedilol, the medium with pH 6.8 is also more biorelevant as a characteristic of intestinal environment where the absorption takes place [48]. Here, the slower dissolution rate and possibly incomplete release was expected due to carvedilol’s physio-chemical properties. Nevertheless, all SMEDDS granules showed complete drug release in this medium as well (>98%), apart from G_m_ SYL_3050_, where 89% of carvedilol was released. Incomplete desorption from solid SMEDDS was reported before, so it is possible that irreversible adsorption was the reason for the incomplete carvedilol release in this case [35]. Finally, the carvedilol release rate was the fastest in G_m_ SYL_224_, where complete drug release was observed within the first 10 min. This could be attributed to the smaller carrier particles with a large specific surface and thus a larger wetting surface area (these granules exhibited the smallest d_50_ value as well). 

The rate and extent of carvedilol release were not affected by the production of SMEDDS granules in a HS granulator with Neusilin^®^ US2, compared to the manual granulation. In vitro dissolution profiles were comparable in both media, with G_m_ NEU releasing 85% of the drug after 5 min, in comparison to 83% for G_hs_ NEU (pH 6.8). On the other hand, when comparing Syloid^®^ 244FP-based granules, the results were somewhat different. In both media, the drug was released more slowly from HS-produced SMEDDS granules, despite the smaller particle size (d_50_ value was 366 µm in comparison to 488 µm, for G_m_ SYL_224_). In support of that, after 5 min in the medium with pH 1.2, 80% of carvedilol was released from G_hs_ SYL_224_, while in the case of G_m_ SYL_224_ this value was 98%. Presumably, due to the high shear forces obtained in the granulator, the liquid was pushed deeper into the pores, resulting in slower release. 

**Figure 7:**
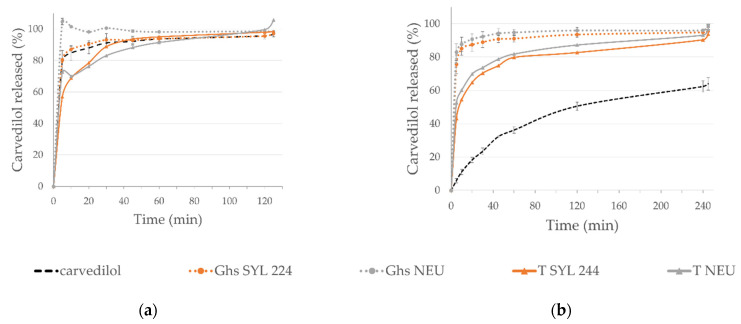
In vitro carvedilol release profiles in dissolution media with pH: (**a**) 1.2; (**b**) 6.8; for SMEDDS granules produced in HS granulator in comparison to corresponding SMEDDS tablets.

Regarding the SMEDDS tablets’ in vitro release profiles (as seen in Figure 7), carvedilol was released slower from tablets than the corresponding granules. When comparing the first 30 min of dissolution testing in a medium with pH 6.8, 92% of carvedilol was released from G_hs_ NEU, while only 74% from corresponding tablets. In case of Syloid^®^ 244FP, the values were 89% (for G_hs_ SYL_244_) vs. 70% (T-SYL_244_). The slower release from tablets can be explained by the compression force breaking the carriers’ porous structure and therefore blocking the pores loaded with SMEDDS [11]. However, the extent of released carvedilol was not impaired with compaction, as the entire dose was released from tablets in the end.

In summary, Neusilin^®^ US2 was determined to be the best mesoporous carrier for further research, as it stands out regarding the dissolution properties of both SMEDDS granules and tablets. Interestingly, Neusilin^®^ US2 was also chosen as the most suitable carrier (among Aeroperl^®^ 300, Zeopharm^®^ 177 and 5170) for SMEDDS tablet preparation in the study of Mura et al., whose result indicated the superiority of SMEDDS tablets over commercial glyburide tables [24].

Overall, concluding from the results presented, carvedilol solubility improvement was accomplished by the production of SMEDDS granule using HS wet granulation, verified by adequate self-microemulsifying properties and followed by the evident improvement of its dissolution behavior, in terms of the release rate and extent of carvedilol released.

## 4. Conclusions

Our research demonstrated that by utilizing HS wet granulation, solid SMEDDS with promising flow properties can be obtained. Liquid SMEDDS was successfully transformed into solid particles using five mesoporous carriers (Syloid^®^ 244FP, Neusilin^®^ US2, Fujicalin^®^ SG, Syloid^®^ XDP 3050 and Aeroperl^®^ 300).

With regard to the amount of SMEDDS incorporated in granules prepared by manual wet granulation (almost 2/3 of granules weight were SMEDDS) and their flow properties, Neusilin^®^ US2 and Syloid^®^ 244FP were chosen as the most promising for granulation using HS granulator, which further improved their flow properties. 

All SMEDDS granules showed improved dissolution properties of carvedilol. In more discriminatory medium with a pH of 6.8, the release of carvedilol was significantly increased by incorporating carvedilol into SMEDDS: for G_m_ SYL_244_, 93% of the drug was released within the first 5 min (with complete release in next 5 min), while for G_m_ NEU, it was 85%. In the case of Neusilin^®^ US2, the release behavior of carvedilol was not affected by the granulation technique, unlike Syloid^®^ 244FP, where the release was slower from HS-produced granules, despite G_m_ SYL_244_ having bigger particle size.

Free-flowing SMEDDS granules produced using high-shear granulator enabled non-problematic die filling and compaction into self-microemulsifying tablets. To ensure adequate hardness, the granules content of the compression mixture had to be reduced to ¼ of total weight, which resulted in a final tablet weight of around 845 mg. Both tablets’ formulations produced from most appropriate granules exhibited fast and complete drug release in both media. Furthermore, all granules and tablets preserved self-microemulsifying properties upon solidification.

Overall, regarding the flow and dissolution properties, Neusilin^®^ US2 stands out as the best mesoporous carrier for further research. SMEDDS tablet formulation optimization would also be of value, in terms of reducing tablet weight containing a single dose of carvedilol, while maintaining appropriate mechanical characteristics. Finally, it would be interesting to address in vitro lipolysis testing (stimulation of digestion), as drug release from SMEDDS formulation is conditioned by the enzymatic degradation of its components. 

## Figures and Tables

**Table 1:** Process parameters set on HS granulator.

Mesoporous Carrier	Impeller Speed (rpm)	Chopper Speed (rpm)
**Syloid^®^ 244FP**	400	2000
**Neusilin^®^ US2**	500	2000
Liquid flow of GD was 4.3 g/min		

**Table 2:** Highest SMEDDS loading and composition of optimal SMEDDS formulations obtained from each mesoporous carrier by either manual (G_m_) or high-shear granulation (G_hs_); granulation dispersion (GD) consisted of various amount of povidone K25 (binder) dissolved in liquid phase composed of 70% SMEDDS and 30% water.

Formulation Name	Mesoporous Carrier	GD Added (g) per 1 g of the Carrier	% Povidone K25 in GD	Liquid Added (g) per 1 g of the Carrier	% SMEDDS in Dry Granules	Process Yield (%)	Moisture Content (%)
G_m_ SYL_244_	Syloid^®^ 244FP	2.87	7	2.68	63.7	90.4	2.18
G_m_ NEU	Neusilin^®^ US2	3.27	7	3.03	66.3	77.2	2.87
G_m_ FUJ	Fujicalin^®^ SG	0.92	5	0.88	36.9	89.5	2.26
G_m_ SYL_3050_	Syloid^®^ XDP 3050	2.56	25	1.93	44.3	89.4	2.11
G_m_ AER	Aeroperl^®^ 300	2.51	15	2.14	53.1	87.6	2.61
G_hs_ SYL_244_	Syloid^®^ 244FP	2.33	7	2.18	59.7	94.2	2.71
G_hs_ NEU	Neusilin^®^ US2	2.27	7	2.12	59.3	73.9	4.18

**Table 3:** Results of SMEDDS granules characterization: size (d_50_), size distribution (SPAN) and flow properties (angle of repose, flow time and Carr index).

Sample	d_50_ (µm)	SPAN	Flow Properties		
			Angle of Repose (°)	Flow Time (s)	Carr Index (%)
G_m_ SYL_244_	448	1.85	18.0 ± 1.7	8.8 ± 0.3	19.2 ± 4.2
G_m_ NEU	529	1.22	24.3 ± 1.4	12.5 ± 1.3	22.9 ± 1.8
G_m_ FUJ	609	0.93	25.4 ± 0.7	11.7 ± 0.4	18.4 ± 1.0
G_m_ SYL_3050_	791	1.25	21.4 ± 1.3	13.5 ± 1.2	35.7 ± 1.5
G_m_ AER	623	0.84	16.7 ± 1.5	7.9 ± 0.2	21.4 ± 0.5
G_hs_ SYL_244_	366	1.90	20.5 ± 2.3	5.8 ± 0.4	15.9 ± 0.9
G_hs_ NEU	329	1.85	25.3 ± 1.4	9.6 ± 0.5	16.4 ± 0.5

**Table 4:** Composition of tableting mixtures during formulation development, with the addition of compression force used and achieved tablet hardness for both most promising carrier types.

	Composition % *w*/*w* *			Syloid^®^ 244FP		Neusilin^®^ US2	
Tableting Mixture	G_hs_ NEU/SYL_244_	Kollidon^®^ VA64	Ac-Di-Sol^®^	Compression Force (kN)	Hardness (N)	Compression Force (kN)	Hardness (N)
TM_0_	100	0	0	15.5	13.0	14.9	24.2
TM_1_	50	5	5	17.4	34.9	15.1	34.2
TM_2_	50	10	5	16.3	35.4	15.1	37.3
TM_3_	50	5	3	15.1	32.8	15.4	39.5
TM_4_	25	5	5	9.5	94.8	9.5	121.5
				10.9	121.2	10.5	137.4

* Avicel^®^ PH-200 was added as a filler up to 100% *w*/*w*.

**Table 5:** Average droplet size (z-average diameter), individual peaks and PDI values of liquid SMEDDS, liquid carvedilol-loaded SMEDDS, granules G_m_ SYL_244_, G_m_ NEU, G_m_ FUJ, G_m_ AER, G_m_ SYL_3050_, G_hs_ SYL_244_ and G_hs_ NEU and tablets with G_hs_ SYL_244_ (T-SYL_244_) and tablets with G_hs_ NEU (T-NEU), after redispersion in three media (water, media with pH 1.2 and pH 6.8).

Sample	Media	z-Average Diameter (nm)	Peak 1 (nm)	Peak 2 (nm)	PDI
**SMEDDS dispersion (without carvedilol)**	water	23.1 ± 0.2	23.1	/	0.07 ± 0.00
	pH = 1.2	25.8 ± 1.6	25.4	2139	0.27 ± 0.05
	pH = 6.8	23.7 ± 0.0	25.1	4293	0.20 ± 0.01
**SMEDDS dispersion** **(with carvedilol)**	water	81.6 ± 1.2	21.9	149.4	0.46 ± 0.00
	pH = 1.2	19.2 ± 0.2	20.9	4576	0.14 ± 0.02
	pH = 6.8	30.9 ± 0.0	34.3	747.6	0.23 ± 0.02
**G_m_ SYL_244_**	water	27.2 ± 0.2	28.1	248.8	0.32 ± 0.00
	pH = 1.2	28.4 ± 0.2	24.0	71.0	0.37 ± 0.00
	pH = 6.8	41.2 ± 0.2	33.7	139.7	0.37 ± 0.01
**G_m_ NEU**	water	21.8 ± 0.0	23.3	4155	0.19 ± 0.01
	pH = 1.2	23.6 ± 0.3	24.3	1343	0.24 ± 0.01
	pH = 6.8	24.7 ± 0.1	26.1	3564	0.21 ± 0.01
**G_m_ FUJ**	water	19.7 ± 0.0	22.1	/	0.13 ± 0.00
	pH = 1.2	25.5 ± 0.2	26.2	332.2	0.28 ± 0.02
	pH = 6.8	30.3 ± 0.0	39.5	2992	0.28 ± 0.02
**G_m_ SYL_3050_**	water	19.9 ± 0.1	23.0	4255	0.16 ± 0.01
	pH = 1.2	40.5 ± 0.5	23.1	142.9	0.47 ± 0.01
	pH = 6.8	35.4 ± 0.2	27.0	181.9	0.43 ± 0.01
**G_m_ AER**	water	22.1 ± 0.1	22.9	1254	0.26 ± 0.01
	pH = 1.2	28.4 ± 0.2	24.7	200.4	0.38 ± 0.01
	pH = 6.8	25.7 ± 0.2	26.5	741.1	0.25 ± 0.00
**G_hs_ SYL_244_**	water	44.9 ± 0.4	29.1	152.8	0.47 ± 0.02
	pH = 1.2	25.2 ± 0.1	23.8	250.2	0.31 ± 0.01
	pH = 6.8	56.8 ± 0.3	27.6	125.9	0.42 ± 0.00
**G_hs_ NEU**	water	22.8 ± 0.0	22.4	3992	0.20 ± 0.01
	pH = 1.2	22.4 ± 0.1	23.9	4442	0.18 ± 0.00
	pH = 6.8	25.1 ± 0.2	27.2	3652	0.21 ± 0.01
**T-SYL_244_**	water	72.2 ± 0.4	25.5	151.4	0.50 ± 0.00
	pH = 1.2	55.4 ± 0.4	22.7	175.8	0.57 ± 0.00
	pH = 6.8	74.6 ± 0.9	26.9	169.2	0.52 ± 0.02
**T-NEU**	water	78.0 ± 1.1	26.1	218.8	0.62 ± 0.02
	pH = 1.2	36.0 ± 1.6	56.1	4454	0.33 ± 0.02
	pH = 6.8	45.1 ± 0.4	34.4	175.5	0.44 ± 0.00

## Data Availability

Not applicable.

## Supplementary Materials

The following supporting information can be downloaded at: https://www.mdpi.com/article/10.3390/pharmaceutics14102077/s1, Table S1: The results of SMEDDS granules characterization during formulation development: GD added per 6 g of carrier, % of povidone K25, d_50_—median particle diameter, SPAN—particle size distribution and granules flow rate (expressed per 100 g); Table S2: Viscosity of GD used for preparation of optimal products with corresponding mesoporous carriers, determined at shear rate of 1 s^−1^. GD consisted of SMEDDS dispersion (70% SMEDDS and 30% water) and binder povidone K25; Figure S1: Viscosity of GD used for preparation of optimal products with corresponding mesoporous carriers, depending on the type of mesoporous carrier used; Figure S2: Particle size distribution of each mesoporous carrier, granules produces manually and in HS granulator, with Syloid^®^ 244FP and Neusilin^®^ US2; Figure S3: SEM images of G_m_ SYL_244_: (a) under magnification 200×; (b) under magnification 1000×; Figure S4: SEM images of G_hs_ SYL_244_: (a) under magnification 200×; (b) under magnification 1000×; Figure S5: SEM images of G_m_ FUJ: (a) under magnification 200×; (b) under magnification 1000×; Figure S6: SEM images of AER: (a) under magnification 200×; (b) under magnification 1000×; Figure S7: SEM images of G_m_ SYL_3050_: (a) under magnification 200×; (b) under magnification 1000×.
