# Supplementary material for: High-Shear Wet Granulation of SMEDDS Based on Mesoporous Carriers for Improved Carvedilol Solubility
Source: Pharmaceutics. 2022 Sep 29;14(10):2077. doi: 10.3390/pharmaceutics14102077 (PMC9606924; doi:10.3390/pharmaceutics14102077)
Supplement: Supplementary file 1 [file pharmaceutics-14-02077-s001.zip › pharmaceutics-1917706-SI.pdf]

pharmaceutics

MDPI

Supplementary Materials: High-Shear Wet Granulation of SMEDDS Based on Mesoporous Carriers for Improved Carvedilol Solubility

Mila Kovačević, Ilija German Ilić, Katarina Bolko Seljak and Alenka Zvonar Pobirk

Table S1. The results of SMEDDS granules characterization during formulation development: GD added per 6 g og carrier, % of povidone K25, d_{50} - median particle diameter, SPAN – particle size distribution and granules flow rate (expressed per 100g).

Mesoporous carrier	GD added per 6 g of carrier (g)	% povidone K25 in GD	d_{50} (μm)	SPAN	Flow time (s)
Syloid® 244FP	15.19	2	108	7.4	7.4
	15.44	4	159	5.2	5.2
	15.56	6	314	5.7	5.7
	17.29	7	448	8.8	8.8
	15.48	10	678	8.9	8.9
	18.82	12	145	8.9	8.9
	15.9	13	452	/	/
	15.88	16	361	/	/
	16.54	18	716	5.9	5.9
Neusilin® US2	19.7	6	330	1.95	9.9
	19.72	7	529	1.22	12.5
	19.09	8	165	1.6	7.8
	18.08	10	219	2.53	9.2
	19.09	12	189	2.46	7.2
	19.45	15	360	1.96	18.4
	20.27	20	602	1.46	16.1
Fujicalin® SG	13.25*	2	266	2.33	6.2
	13*	3	233	2.81	4.8
	16.35*	4	437	1.19	7.15
	18.5*	5	609	0.93	11.7
	13.55*	7	410	1.88	6.0
	12.93*	10	751	0.47	5.9
Syloid® XDP 3050	13.77	5	58.8	0.97	7.0
	13.63	6	56.4	0.88	7.2
	14.83	7	60.9	0.94	∞
	15.48	10	106	2.39	/
	15.72	15	351	1.78	26.7
	15.65	17	340	1.66	/
	15.62	20	723	1.18	15.8
	15.45	25	791	1.25	13.5
	15.38	30	879	1.06	/
Aeroperl® 300	15.25	10	332	2.56	8.4
	15.23	15	623	0.84	7.9
	15.58	17	710	0.78	9.1
	15.62	20	602	0.93	16.1

*GD added per 20 g of carrier (g).

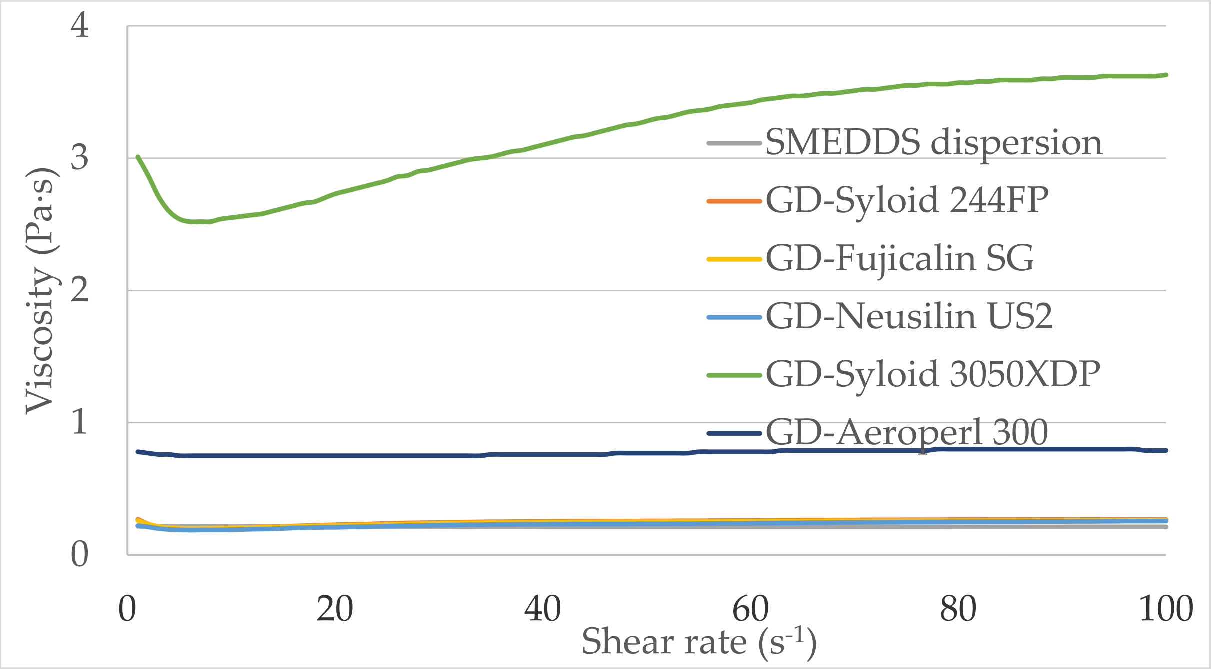

Figure S1. Viscosity of GD used for preparation of optimal products with corresponding mesoporous carriers, depending on the type of mesoporous carrier used. .

Table S2. Viscosity of GD used for preparation of optimal products with corresponding mesoporous carriers, determined at shear rate of 1 s^{-1}. GD consisted of SMEDDS dispersion (70 % SMEDDS and 30 % water) and binder povidone K25.

Sample	% povidone K25	Viscosity (Pa·s)
SMEDDS dispersion	0	0.62
GD-Syloid® 244FP	7	0.67
GD-Neusilin® US2	7	0.62
GD-Fujicalin® SG	5	0.66
GD-Syloid® 3050 XDP	25	3.41
GD-Aeroperl® 300	15	1.18

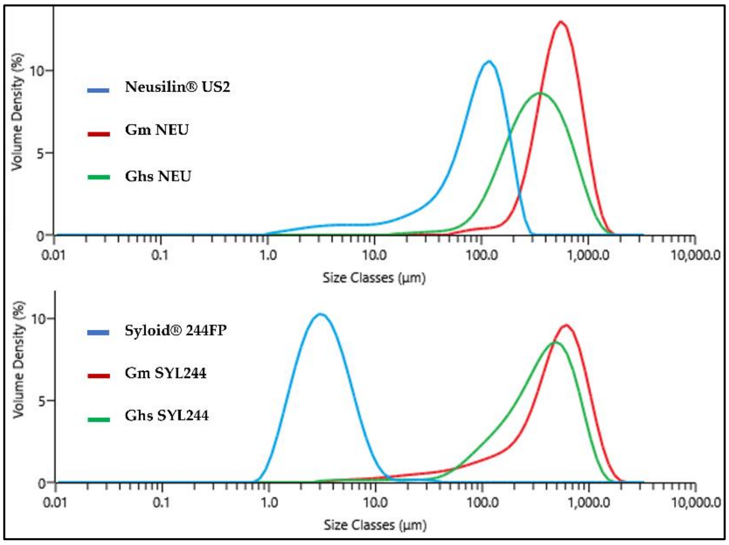

Figure S2. Particle size distribution of each mesoporous carrier, granules produces manually and in HS granulator, with Syloid® 244FP and Neusilin® US2.

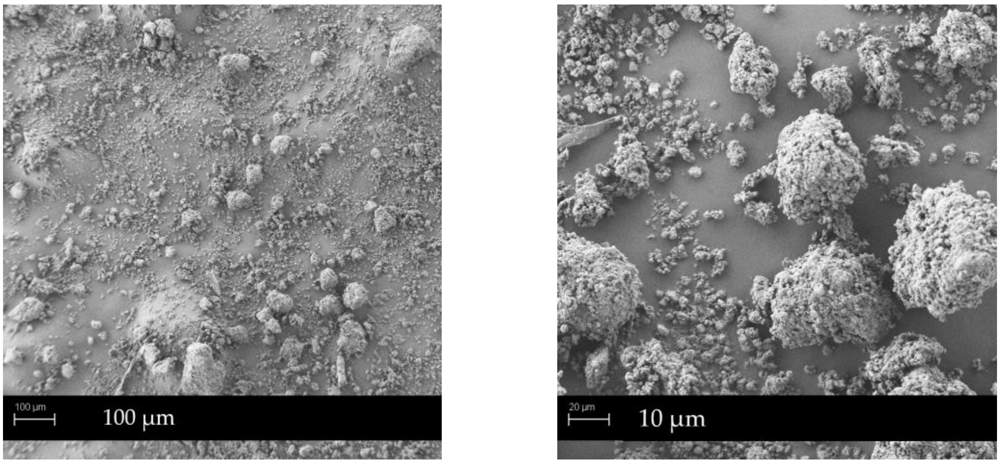

(**a**) (**b**)

Figure S3. SEM images of G_m SYL_{244}: (**a**) under magnification 200x; (**b**) under magnification 1000x.

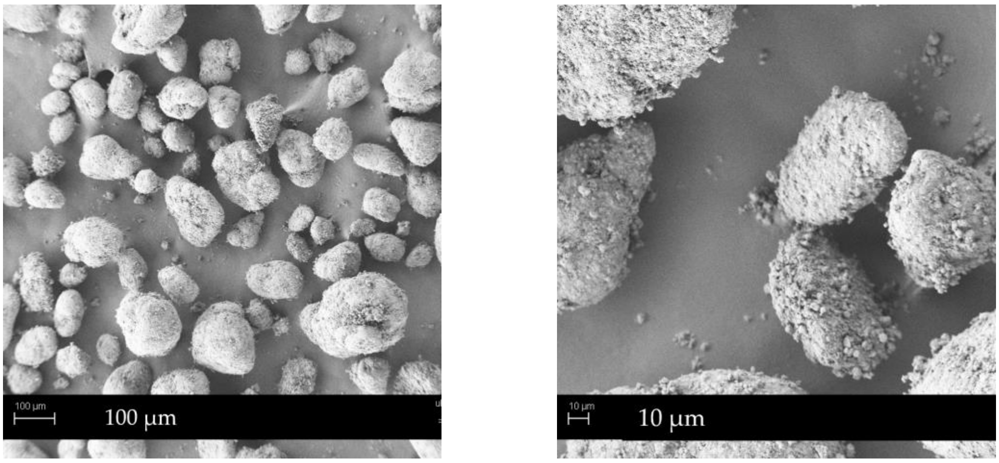

(**a**) (**b**)

Figure S4. SEM images of G_{hs} SYL_{244}: (**a**) under magnification 200x; (**b**) under magnification 1000x.

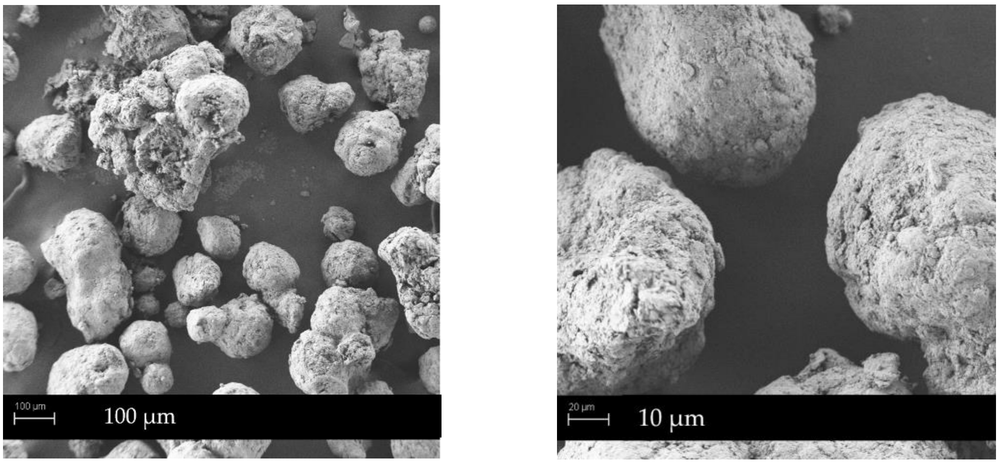

(**a**) (**b**)

Figure S5. SEM images of G_m FUJ: (**a**) under magnification 200x; (**b**) under magnification 1000x.

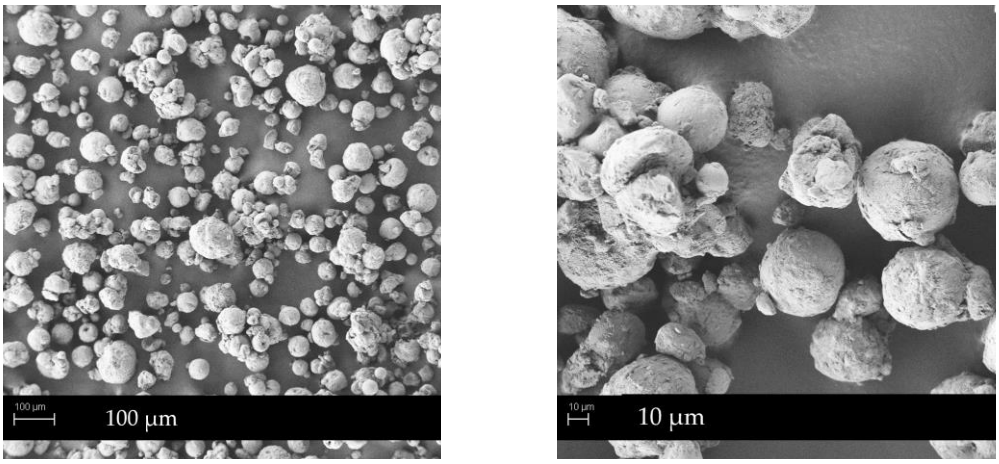

(**a**) (**b**)

Figure S6. SEM images of G_m AER: (**a**) under magnification 200x; (**b**) under magnification 1000x.

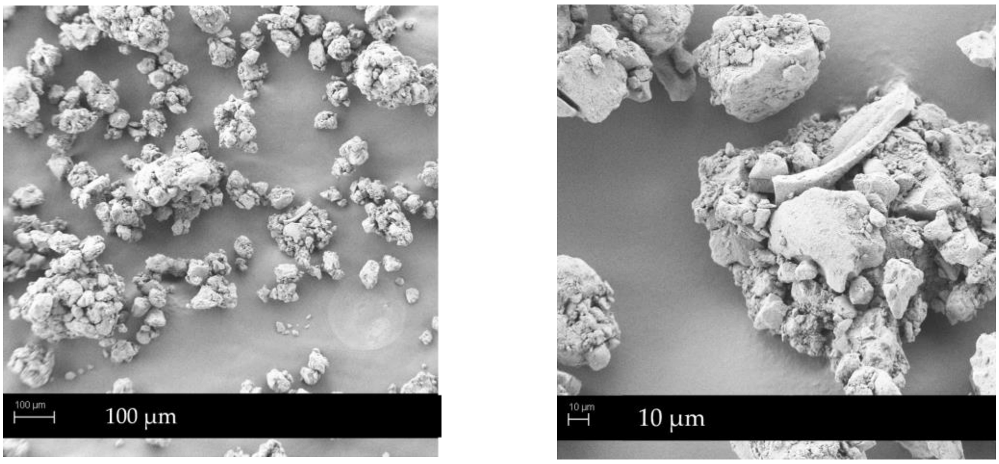

(**a**) (**b**)

Figure S7. SEM images of G_m SYL_{3050}: (**a**) under magnification 200x; (**b**) under magnification 1000x.
